# No changes in triple network engagement following (combined) noradrenergic and glucocorticoid stimulation in healthy men

**DOI:** 10.1093/scan/nsad073

**Published:** 2023-12-20

**Authors:** Renée Lipka, Catarina Rosada, Sophie Metz, Julian Hellmann-Regen, Hauke Heekeren, Katja Wingenfeld

**Affiliations:** Charité—Universitätsmedizin Berlin, Department of Psychiatry and Neurosciences, Campus Benjamin Franklin, Berlin 12203, Germany; Humboldt Universität zu Berlin, Berlin School of Mind and Brain, Berlin 10099, Germany; Charité—Universitätsmedizin Berlin, Department of Psychiatry and Neurosciences, Campus Benjamin Franklin, Berlin 12203, Germany; Charité—Universitätsmedizin Berlin, Department of Psychiatry and Neurosciences, Campus Benjamin Franklin, Berlin 12203, Germany; Charité—Universitätsmedizin Berlin, Institute of Medical Psychology, Campus Mitte, Berlin 10117, Germany; Charité—Universitätsmedizin Berlin, Department of Psychiatry and Neurosciences, Campus Benjamin Franklin, Berlin 12203, Germany; Universität Hamburg, Executive University Board, Hamburg 20148, Germany; Charité—Universitätsmedizin Berlin, Department of Psychiatry and Neurosciences, Campus Benjamin Franklin, Berlin 12203, Germany

**Keywords:** triple network, salience network, default mode network, executive control network, stress neuromodulators

## Abstract

Successful recovery from stress is integral for adaptive responding to the environment. At a cellular level, this involves (slow genomic) actions of cortisol, which alter or reverse rapid effects of noradrenaline and cortisol associated with acute stress. At the network scale, stress recovery is less well understood but assumed to involve changes within salience-, executive control-, and default mode networks. To date, few studies have investigated this phase and directly tested these assumptions. Here, we present results from a double-blind, placebo-controlled, between-group paradigm (*N *= 165 healthy males) administering 10 mg oral yohimbine and/or 10 mg oral hydrocortisone two hours prior to resting state scanning. We found no changes in within-network connectivity of the three networks, both after single and combined drug administration. We further report the results of Bayesian parameter inference to provide evidence for the null hypothesis. Our results contrast with previous findings, which may be attributable to systematic differences between paradigms, highlighting the need to isolate paradigm-specific effects from those related to stress.

## Introduction

Once a threat is detected, two neuromodulatory systems become active. First, the nucleus coeruleus shifts from phasic bursts towards tonic high-frequency firing, increasing noradrenaline release along its widespread (sub)cortical projections (Valentino and Van Bockstaele, 2008). By binding to low-affinity α1 and β adrenergic receptors, which often have opposing effects, noradrenaline overarchingly increases the brain’s signal-to-noise ratio—enhancing responsiveness to salient information while dampening most other activity (O’Donnell *et al.*, 2008). Around the same time, activity along the hypothalamic-pituitary-adrenal axis, involving a cascade of hormones and neuropeptides, eventually leads to the release of glucocorticoid hormones from the adrenal cortex. The predominant glucocorticoid in humans is cortisol. Cortisol readily passes the blood brain barrier and, once in the brain, binds to mineralocorticoid receptors (MRs) and glucocorticoid receptors (GRs) (Joëls, 2018). The MR has high affinity for glucocorticoids and is therefore occupied as soon as cortisol first reaches the brain (15–30 minutes following noradrenaline) (Joëls and Baram, 2009). These early effects of cortisol then conspire with noradrenaline to shift the organism into a ‘fight or flight’ mode—increasing vigilance, mobilizing glucose stores, and promoting its distribution through increases in heart rate and blood pressure (de Kloet, 2014). After approximately one hour, noradrenaline levels return to baseline, while cortisol has amassed to levels sufficient for binding to lower-affinity GRs. GRs are involved in the termination of the stress response and (mainly) induce slower genomic effects. Genomic cortisol actions take at least one hour to initiate, but last from hours to days (Joëls, 2018). Overall, the stress response can be divided into two broad phases: one acute emergency phase (primarily mediated by adrenergic and MR binding) and one recovery and priming phase (primarily mediated by GR). For an optimal response to stressors, the functioning of both phases is equally important (de Kloet *et al.*, 2018; Joëls, 2018).

Moving to a coarser resolution of the brain, cellular surges in stress neuromodulators express as dynamic shifts in large-scale neural networks (Hermans *et al.*, 2014). These networks are known to act as functional units, characterized by temporal correlations between constituent brain regions, both while at rest and during task performance (Smith *et al.*, 2009). In the context of stress, the networks which have received most attention are summarized in the ‘triple network model’ (Menon, 2011; van Oort *et al.*, 2017). The first network included in the model is termed the executive control network (ECN). The ECN is a fronto-parietal system, centered on the dorsolateral prefrontal cortex and lateral posterior parietal cortex, which exhibits strong intrinsic connectivity during many cognitively demanding tasks (Menon, 2011; Menon and D’Esposito, 2022). The second network, the default mode network (DMN), is a medial temporal system composed of core nodes in the posterior cingulate cortex, medial prefrontal cortex, medial temporal lobe and angular gyrus (Menon, 2023). The DMN is commonly associated with social cognition, self-referential processing, mind wandering, and memory. In contrast to the ECN, the DMN is largely deactivated during cognitive tasks, except for when these tasks require self-generated internal cognition (e.g. recalling autobiographical memories or inferring the mental states of others) (Menon, 2023). The final network is termed salience network (SN), a system anchored in the dorsal anterior cingulate and fronto-insular cortex, which further includes the amygdala, and has been implicated in promoting alertness, attentional (re)orienting, and vigilance (Menon, 2011; Menon and D’Esposito, 2022). Upon detection of a salient stimulus, the triple network model places the SN in a delegating position, rapidly reorienting attention and initiating network switches depending on task demands: if externally directed cognition is required, the SN recruits the ECN and disengages the DMN. Conversely, when internally directed cognition is required, the SN engages the DMN while uncoupling the ECN (Menon, 2011, 2023). Stress neuromodulators affect all of these networks, though in temporally specific and often opposing ways.

In the context of acute stress (immediately surrounding stressor onset), van Oort *et al.,* (2017) have systematically reviewed the effects of several types of experimental stress induction on triple network changes. This included exposure to extreme sensory stimulation (excessive cold or loud noises), watching aversive movie material, re-imagining a personally stressful experience, or having to perform a free speech and/or a higher-order cognitive task. The latter methods commonly involve time pressure and a critical audience providing (negative) feedback (van Oort *et al.*, 2017). Perhaps the most consistent finding across paradigms was an increase in activity and connectivity within the SN (e.g. Hermans *et al.*, 2011; van Leeuwen *et al.*, 2021) and DMN (e.g. Sinha *et al.*, 2004; Vaisvaser *et al.*, 2013). The ECN showed no changes across the majority of studies (e.g. Clemens *et al.*, 2017; van Leeuwen *et al.*, 2021), but some evidence suggests that its functions may share an inverted U-shape function with stress. That is, cognitive performance may be boosted at intermediate levels of stress neuromodulators, but break down at higher levels (van Oort *et al.*, 2017).

Compared to the acute phase surrounding stressor onset, the neural signature describing an adaptive recovery from stress (one hour after stressor onset and beyond) is not well understood (van Oort *et al.*, 2017). This is despite a body of evidence indicating that this phase may be more than a mere return to baseline, but serves its own adaptive niche by dampening emotional reactivity and promoting cognitive functions which prepare the organism for the future (Hermans *et al.*, 2014; de Kloet *et al.*, 2018; Joëls, 2018). For example, 75 min following hydrocortisone (synthetic cortisol) administration, amygdala (an SN node) reactivity to emotional faces was dampened (Henckens *et al.*, 2010). Even later, 240 minutes after hydrocortisone intake, participants’ working memory performance was significantly increased, and this was accompanied by increased activity in the dorsolateral prefrontal cortex (an ECN node) (Henckens *et al.*, 2011). Based on such findings, Hermans *et al.,* (2014) predicted stress-induced network changes to be actively reversed within the second hour following stress, that is, an SN down- and ECN upregulation (Hermans *et al.*, 2014). These predictions were partly endorsed by findings of reduced SN connectivity and increased ECN-cerebellum connectivity 90 minutes following psychosocial stress induction (van Leeuwen *et al.*, 2021). No predictions were made regarding the DMN, although some evidence suggests that there is increased coupling between the amygdala (SN node) and several DMN regions up to 2 hours following psychosocial stress (Veer *et al.*, 2011; Vaisvaser *et al.*, 2013). So far, it is unclear whether this relates to adaptive processes of emotion regulation and memory consolidation (Veer *et al.*, 2011) or marks vulnerability of those who did not secrete sufficient cortisol to initiate the GR-mediated termination signal (Vaisvaser *et al.*, 2013).

There are two important methodological considerations regarding investigations of the stress recovery phase. (1) A number of studies have administered hydrocortisone in the absence of other neuromodulators. Though this is sensible from the perspective that cellular effects of this phase should mainly be governed by (genomic) actions of cortisol, it disregards that actions of stress neuromodulators are linked in complex preparatory and suppressive ways (Joëls and Baram, 2009) which limits the ecological validity of these findings (Harrewijn *et al.*, 2020). (2) All but one study (van Leeuwen *et al.*, 2021) that we know of have focused on single regions, investigating either activity when comparing stress and control conditions, or connectivity between selected regions of interest (seeds) and a small number of temporally correlated regions. Such approaches have potentially overlooked important network changes outside of the investigated areas.

In summary, two main neuromodulatory systems (noradrenaline and cortisol) coordinate two broad phases of the stress response (acute and recovery). During acute stress, heightened levels noradrenaline and cortisol are associated with well replicated shifts towards the SN and DMN, which may come at the cost of the ECN. During recovery from stress, when noradrenaline levels normalize and cortisol starts exerting slower genomic (GR mediated) actions, current evidence points towards a downregulation of the amygdala (SN node), accompanied by an upregulation of the dorsolateral prefrontal cortex (ECN node), and increased connectivity between the amygdala and nodes of the DMN. To date, the recovery phase remains poorly understood at the network scale, as most paradigms have focused on single regions or administered hydrocortisone without concurrent noradrenergic stimulation.

The aim of the current study was to investigate the delayed effects of both noradrenaline and cortisol on the SN, DMN, and ECN from a whole network perspective. We employed a pharmacological paradigm administering both oral yohimbine (which increases noradrenergic activity) and/or hydrocortisone, in order to observe their effects on resting state connectivity two hours following drug intake, a timeframe consistent with the early stress recovery phase. In line with previous predictions of this phase, we expected pharmacological elevation of noradrenaline and cortisol (especially in their combination) to lead to increases in ECN connectivity and decreases in SN connectivity (as compared to placebo). Since the evidence is equivocal as to whether an adaptive recovery from stress would involve a DMN up- or downregulation, we explored DMN connectivity changes in both directions.

## Materials and methods

### Design

We employed a randomized, double-blind, placebo-controlled, cross-over design in order to investigate the influence of 10 mg of oral yohimbine and/or 10 mg of oral hydrocortisone on resting state connectivity within the ECN, DMN, and SN. Participants were randomly assigned to one of four treatment groups: (A) yohimbine + placebo, (B) placebo + hydrocortisone, (C) yohimbine + hydrocortisone and (D) placebo + placebo. Yohimbine and cortisol dosage was aligned with other pharmacological paradigms investigating healthy males (e.g. Henckens *et al.*, 2010; Chae *et al.*, 2019). Since cellular and network changes related to stress recovery theoretically begin one hour after stressor onset and last for several hours (Hermans *et al.*, 2014), our timepoint was chosen to both reflect this interval (two hours following medication intake) while also reducing participant burden of lying in the scanner for more than 80 minutes. Drug timing was chosen such that noradrenaline and cortisol would reach peak plasma levels (Garrard, 2014; Hindmarsh and Geertsma, 2017) when participants first entered the scanner. Although other tasks of the study were preregistered (https://osf.io/j53f7; https://osf.io/d2mct/), the resting state data used in the current work was not. Our analyses should therefore be considered exploratory.

### Participants

Sample size was computed a priori on the basis of group level statistical maps of our previous study administering 10 mg of hydrocortisone (Metz *et al.*, 2019) using neuropower (http://neuropowertools.org/; now discontinued), which showed that for a power of 0.80, a sample size of *N* = 37 per group would be sufficient (we increased this number by 10% in order to account for potential drop outs). Recruitment took place through the hospital website and social media advertisements. In total, 167 healthy male volunteers aged 18–35 were recruited. Exclusion criteria were self-reported history of physical or psychiatric illness, self-reported history of trauma, current medication intake, lifetime history of drug use or alcohol dependence, shift work, a body mass index > 30, left-handedness, and other Magnetic Resonance Imaging (MRI) contraindicators. Participants were compensated with either student credit or a value between 60 and 90€ (depending on the performance during a decision making task). The study was conducted in accordance with the Declaration of Helsinki and was approved by the medical ethics committee of the Charité Universitätsmedizin Berlin. All participants gave written informed consent. Two participants were excluded due to premature termination of the experiment or artifactual resting state data. In total, data from 165 participants were analyzed: yohimbine + placebo (*N* = 40), placebo + hydrocortisone (*N* = 41), yohimbine and hydrocortisone (*N* = 42), and placebo + placebo (*N *= 42). Blinding was confirmed by letting participants and experimenters guess whether a drug or placebo had been taken. Participants performed below chance (39% correct) and experimenters performed at chance level (50% correct).

### Procedure

Participants came for two visits: (1) one screening visit during which they underwent a physical examination and completed a number of clinical questionnaires and (2) an MRI scanning visit. At the scanning day, participants were tested in the afternoon (either at 2.30 or 4.30 pm) and instructed to refrain from exercising, eating or consuming caffeine one hour prior. At arrival, participants were placed in a quiet room to give their first set of physiological samples (blood pressure, heart rate, and saliva; *t* = 0). They then received the experimental instructions and gave a second set of physiological samples (*t* = +15). Afterwards, they received the first drug (yohimbine/placebo; *t* = +15) and after a short delay (*t* = +30) the second drug (hydrocortisone/placebo). At *t* = +75, participants gave another set of physiological samples and were escorted to the scanner room, where they entered the scanner. The scanning protocol comprised multiple scans: a decision making task, a dotprobe task, a resting state scan, and a T1 structural scan. Only the resting scan (*t* = +135) and T1-weighted image (*t* = +145) were utilized for the present study. After scanning, participants gave two final physiological samples (at times *t* + 155 and *t* + 170).

### Physiological data

#### Acquisition

Blood pressure and heart rate were assessed using portable upper arm blood pressure monitors (boso medicus family, Jungingen, Germany). Saliva samples were taken using Salivette cotton swabs (Salivette, Nürbrecht, Germany) and stored at −80°C until biochemical analysis. Saliva was tested for concentrations of cortisol and alpha amylase (a marker of adrenergic activity; Vanstegeren *et al.*, 2006) at the neurobiological laboratory of Charité Universitätsmedizin, Campus Benjamin Franklin. Alpha amylase was assessed using a direct alpha amylase assay using 2-chloro-4-nitrophenyl-a-D-maltotrioside and free cortisol was assessed using an adapted homogeneous time-resolved fluorescence resonance energy transfer-based competitive immunoassay (for details see Lorentz *et al.*, 1999; Ehlert *et al.*, 2006).

#### Data analysis

Baseline characteristics of treatment groups were investigated using one-way between subjects’ analyses of variance (ANOVAs) for continuous variables and Chi square tests for categorical variables. Manipulation checks were carried out using mixed repeated measures ANOVAs, with treatment variables yohimbine (yes/no) and hydrocortisone (yes/no) as between subjects factors and time as the within subjects factor. Significant ANOVA F-tests were followed by Bonferroni-corrected independent samples t-tests (to compare means between treatment groups) and Bonferroni-corrected paired samples t-tests (to compare to baseline within treatment groups). Reported effect sizes (ESs) were eta squared for F-tests and Cohen’s d for t-tests. Statistical analyses were carried out using SPSS (v26.0, IBM Armonk, New York).

### MRI data

#### Acquisition

MR data were acquired on a 3T Siemens Magnetom TimTrio with a 32-channel head coil (Siemens Healthcare, Erlangen, Germany). For the resting state scan (10 minutes), participants were instructed to lie still while not thinking about anything in particular and fixating at the center of a black screen. Acquisition parameters for the T2*-weighted echo planar imaging sequence were: field-of-view = 192 mm, flip angle = 70°, slice thickness = 3 mm, inter-slice gap = 3.3 mm, voxel resolution = 3 × 3 × 3 mm, repetition time (TR) = 2000 ms, echo time (TE) = 30 ms, resulting in a total of 300 whole-brain volumes. Acquisition parameters for high resolution T1-weighted structural scans were: field-of-view = 256, flip angle = 9°, slice thickness = 1 mm, interslice gap = 0 mm, voxel resolution 1 × 1 × 1, TR = 1900 ms, and TE = 2.52 ms.

#### Preprocessing

Structural scans were brain extracted using the Advanced Normalization Tools abpBrainExtraction script (Avants *et al.*, 2009). Where necessary, brain extraction was improved by manually editing slices in freeview (v.6.0.0; https://surfer.nmr.mgh.harvard.edu/). All other analysis steps were carried out in FMBRIB’s Software Library (FSL; v 6.0.5; Jenkinson *et al.*, 2012). Structural data were reoriented, bias-field corrected, and segmented into tissue types using the fsl_anat script (https://fsl.fmrib.ox.ac.uk/fsl/fslwiki/fsl_anat). Preprocessing of functional data consisted of brain extraction and bias field correction using BET (Brain Extraction Tool; https://fsl.fmrib.ox.ac.uk/fsl/fslwiki/BET; Smith, 2002), motion correction using MCFLIRT (Motion Correction FMRIB Linear Registration Tool; https://fsl.fmrib.ox.ac.uk/fsl/fslwiki/MCFLIRT; Jenkinson *et al.*, 2002), spatial smoothing (4 mm full width half maximum; https://fsl.fmrib.ox.ac.uk/fsl/fslwiki/SUSAN; Smith and Brady, 1997), co-registration via BBR (Boundary Based Registration; https://fsl.fmrib.ox.ac.uk/fsl/fslwiki/FLIRT_BBR), and normalization into standard space using FNIRT (FMRIB’s Nonlinear Image Registration Tool; https://fsl.fmrib.ox.ac.uk/fsl/fslwiki/FNIRT; Andersson *et al.*, 2007; template = MNI152 T1 2 mm, warp resolution = 10 mm). An analysis of absolute mean displacements confirmed that motion overall was low (*M* = 0.3 mm, SD = 0.56) and that groups did not differ in motion (F(3, 161) = 0.914, *P* = 0.436, *η^2^ *= 0.017). Finally, independent component analysis (ICA) based advanced removal of motion artifacts (ICA-AROMA) was implemented for final denoising of single-subject resting state data (https://fsl.fmrib.ox.ac.uk/fsl/fslwiki/OtherSoftware; Pruim *et al.*, 2015).

#### Network extraction

To identify the networks of interest within our sample, we ran a group ICA on the preprocessed, temporally concatenated resting state data of all participants using MELODIC with 30 components (Multivariate Exploratory Linear Optimized Decomposition into Independent Components; https://fsl.fmrib.ox.ac.uk/fsl/fslwiki/MELODIC; Beckmann and Smith, 2004) . To identify the DMN, ECN, and SN, we ran spatial cross-correlations between our unthresholded group components and component templates for the DMN and ECN provided by Smith and group, as well as spatially concatenated anterior and posterior SN templates provided by Shirer and colleagues (Smith *et al.*, 2009; Shirer *et al.*, 2012). The best-fitting networks were visually inspected by two authors (R.L. and C.R.) and checked to include core regions of each network. To relate group-level components back to individual participants, a dual regression was performed (https://fsl.fmrib.ox.ac.uk/fsl/fslwiki/DualRegression; Nickerson *et al.*, 2017). Dual regression outputs the subject-specific spatial maps for each of the 30 components, which were then used as input for the group comparisons.

#### Group comparisons

To examine whether within network connectivity of medication groups differed from the placebo group, we used FSL’s permutation test randomize, with 10 000 permutations and threshold-free cluster enhancement to correct for multiple comparisons across voxels *within* networks (https://fsl.fmrib.ox.ac.uk/fsl/fslwiki/Randomise/UserGuide; Winkler *et al.*, 2014). We did not include motion, cerebrospinal fluid, or white matter regressors, since such nuisance signal was removed by ICA-AROMA during preprocessing. Pairwise comparisons were made between each treatment group and the placebo group. To correct for multiple comparisons *across* networks, we used Bonferroni correction (adjusted alpha level 0.05/3 = 0.016). Finally, we extracted each individuals’ mean beta estimates for the three networks of interest. This was done by creating masks for each network by thresholding (z ≥ 3) and binarizing the corresponding components acquired during the 30-component group ICA. Then, these masks were used to extract mean beta estimates from each individual’s spatial map of the components of interest, which were acquired during the second step of the dual regression. This resulted in three values per participant, representing an estimate of mean within-network connectivity of the SN, DMN, and ECN.

In order to investigate whether the absence of network differences between groups reflected evidence for the null hypothesis, we additionally conducted group-level Bayesian parameter inference (BPI) in SPM12 (Statistical Parametric Mapping; http://www.fil.ion.ucl.ac.uk/spm; v7771) using the BayInf toolbox (Masharipov *et al.*, 2021). BPI evaluates the posterior probability of finding the experimental effect within or outside the region of practical equivalence (ROPE) to the null value. Parameter estimation of the BayInf toolbox is based on a parametric empirical Bayes approach with a ‘global shrinkage’ prior (Masharipov *et al.*, 2021). For each network, we compared the combined medication group (yohimbine and hydrocortisone) to the placebo group. Parameter inference was done using the ROPE-only decision rule, with the default ES thresholds of one prior standard deviation of the group experimental effect. Estimated ES thresholds were: 0.85 for the DMN, 0.93 for the SN, and 0.79 for the ECN. The posterior probability threshold was *P*_thr_ = 95%, which is equal to Log Posterior Odds (LPO) > 3 (Masharipov *et al.*, 2021). This way, voxels with 95% of their posterior probability distribution outside the ROPE were classified as either showing ‘increased’ or ‘decreased’ network strength, depending on which side of the ROPE the distribution was located. Those voxels with 95% of their posterior probability distribution within the ROPE were considered to show ‘no changes’ in network strength. If none of these criteria were met, voxels were considered ‘low confidence voxels’, for which our data were insufficient to make inferences. A more detailed description of the BPI procedure can be found in the Supplementary Material.

## Results

### Baseline characteristics

To examine whether there were significant differences in baseline participant characteristics across treatment groups, we ran one-way ANOVAs. There were no systematic differences in age, body mass index, level of education (German A-level attainment), or trait anxiety levels (assessed during the physical examination visit). Groups also did not differ in state anxiety levels (assessed just before entering the scanner). The results are summarized in Table 1.

**Table 1.** Sample characteristics

	Medication group					
Characteristic	Placebo	Yohimbine	Hydrocortisone	Yohimbine and hydrocortisone	Statistics	*P*
Age (*M*, SD)	25.36 (4.42)	26.10 (4.05)	24.71 (4.47)	24.88 (4.14)	F(3,161) = 0.85, *η^2^ *= 0.01	0.46
Education (*N*)	39	38	40	40	*Χ^2^*(3) = 0.80, *V *= 0.78	0.80
BMI (*M*, SD)	23.70 (2.79)	23.17 (2.70)	23.22 (2.54)	23.47 (2.24)	F(3,160) = 0.36, *η^2^ *= 0.00	0.77
Trait Anxiety (STAI-T)	34.88 (9.66)	34.90 (8.28)	33.24 (7.95)	30.90 (4.83)	F(3,161) = 2.38, *η^2^ *= 0.04	0.07
State Anxiety (STAI-S)	18.78 (7.07)	20.41 (13.32)	20.34 (12.99)	15.73 (8.72)	F(3,160) = 1.69, *η^2^ *= 0.03	0.17

Abbreviations: BMI = Body Mass Index, STAI = State-Trait Anxiety Inventory. BDI = Beck Depression Inventory. Note: Education refers to number of participants with German A level attainment.

### Salivary alpha amylase and cortisol

To investigate the effect of group assignment on salivary alpha amylase and cortisol, two mixed repeated measures ANOVAs were performed with time as the within-subjects factor and yohimbine (yes/no) and hydrocortisone (yes/no) as between-subjects factors. There was a significant effect of yohimbine on salivary alpha amylase: F(1, 161) = 8.09, *P* = 0.005, *η^2^= 0.*04 and a time × yohimbine interaction: F(3.43, 552.96) = 6.48, *P* < 0.001, *η^2^ = *0.03. Bonferroni corrected post hoc *t*-tests (one tailed) revealed that groups who received yohimbine, as compared to groups who did not, had higher mean levels of salivary alpha amylase at the three timepoints following drug administration: time +75 (t(163) = 3.22, *P* = 0.009, *d* = 0.50), time +155 (t(163) = 3.21, *P* = 0.009, *d* = 0.50), and time +170 (t(163) = 3.33, *P* = 0.006, *d* = 0.51). Moreover, a Bonferroni corrected paired samples *t*-test revealed that at the timepoint which most closely matched the timing of the resting state scan (+155), yohimbine-receiving groups still had significantly elevated levels of salivary alpha amylase (as compared to baseline; t(81) = −4.24, *P* < 0.001, *d* = 0.46). There was no effect of hydrocortisone on salivary alpha amylase: F(1, 161) = 1.36, *P* = 0.245, *η^2^ *= 0.00.

**Fig. 1.**
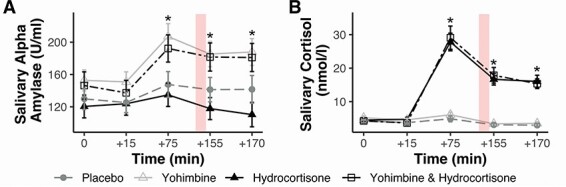
Salivary alpha amylase (A) and salivary cortisol (B) across time. The first drug (10 mg yohimbine/placebo) was administered at time +15, and the second drug (10 mg hydrocortisone/placebo) was administered at time +30. Background shading indicates the time of resting state scanning. Error bars represent standard errors of the mean. * indicates statistically significant differences between groups receiving either yohimbine or hydrocortisone, compared to groups that did not (Bonferroni corrected, at *P* < 0.05).

With regard to salivary cortisol, there was a significant effect of hydrocortisone: F(1, 161) = 97.87, *P *< 0.001, *η^2^ *= 0.37, and a time × hydrocortisone interaction: F(1.64, 265.07) = 74.77, *P* < 0.001, *η^2^ *= 0.31. Bonferroni corrected post-hoc *t*-tests (one tailed) revealed that groups who had received cortisol, compared to those who did not, had higher mean salivary cortisol at the following three timepoints: time +75 (t(97.47) = 9.73, *P* < 0.001, *d* = 1.50), time +155 (t(90.98) = 9.26, *P* < 0.001, *d* = 1.43), and time +170 (t(97.91) = 9.91, *P* < 0.001, *d* = 1.53). Further, Bonferroni corrected paired samples *t*-tests showed that, at the timepoint closest to resting state scanning (+155), groups which received hydrocortisone still had significantly elevated levels of salivary cortisol (when compared to baseline; t(82) = −8.56, *P* < 0.001, *d* = 0.94). There was no effect of yohimbine on salivary cortisol: F(1, 161) = 0.04, *P* = 0.83, *η^2^ *= 0.00. See Figure 1 for an overview of mean levels of salivary alpha amylase and cortisol over time. Additional analyses of heart rate and blood pressure can be found in the Supplementary Material.

### Network connectivity

Network components for the three networks of interest each included commonly cited core nodes. In the case of the SN, core nodes are the anterior cingulate and fronto-insular cortices (Menon and Uddin, 2010); for the DMN, the posterior cingulate, medial prefrontal cortices, and medial temporal lobes (Andrews-Hanna *et al.*, 2015), and for the ECN the dorsolateral prefrontal- and lateral posterior parietal cortices (Menon, 2011). Of note, our network components each included core regions, except for the ECN, which was missing the lateral posterior parietal cortex. Our component can therefore be best described as the anterior ECN. See Supplementary Figure 1 for a visualization of group-level components selected for the analysis.

**Fig. 2.**
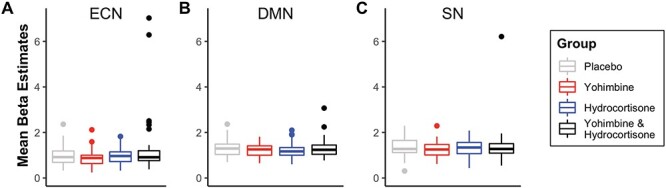
Mean beta estimates across three networks of interest: (A) the ECN, (B) the DMN, and (C) the SN. There were no significant differences in within network connectivity between treatment groups. Whisker plots show first quartiles, median values, and third quartiles. Whiskers denote 1.5 times the interquartile range. Dots denote observations outside this range.

Non-parametric voxel-wise comparisons of connectivity within the SN and DMN revealed no significant differences between treatment groups and the placebo group. For the ECN, the contrast between the combined medication and placebo group showed some significant clusters. Within these clusters, the majority of voxels were located in the cerebral white matter and spread without meaningful coherence—likely reflecting residual noise rather than signal. The results of the group contrasts are summarized in Supplementary Table 1. To visualize results, we extracted mean within network connectivity estimates (beta estimates, one value per network for each participant). The results are shown in Figure 2.

**Fig. 3.**
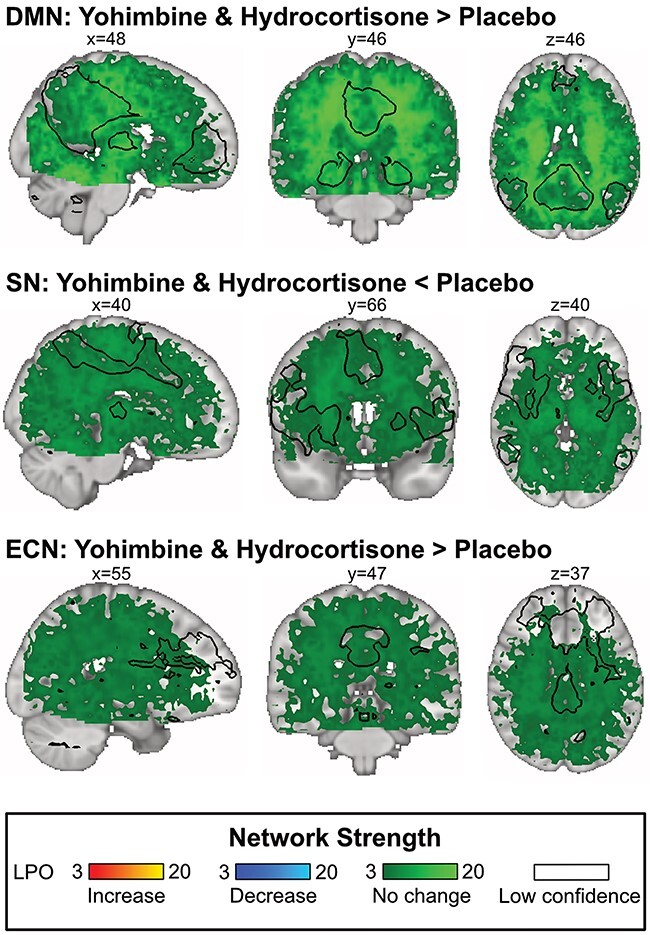
Results of the BPI and network outlines (thresholded at *z* ≥ 3) overlaid over the MNI152 standard space. BPI was implemented using the ‘ROPE-only’ decision rule with ES threshold = one prior SD of the group experimental effect and *P*_thr_ = 95% (LPO > 3). Abbreviations: DMN = Default Mode Network, ECN = Executive Control Network, ES = Effect Size, LPO = Log Posterior Odds, MNI = Montreal Neurological Institute, ROPE = Region Of Practical Equivalence, SD = Standard Deviation, SN = Salience Network.

To investigate whether the absence of network differences between groups reflected evidence for the null hypothesis, we conducted additional group-level BPIs for the combined medication group *vs* the placebo group. Results showed that for each comparison, no voxels were classified as showing ‘increased’ or ‘decreased’ network strength (all LPOs < 3). The majority of voxels were classified as ‘no change’ in network strength (with LPOs ≥ 3 in 77.34% of voxels for the DMN, 62.55% for the SN, and 53.35% for the ECN). The remaining voxels of each comparison were low confidence voxels for which our data were insufficient to make inferences. This provides clear evidence for the null hypothesis for all three network comparisons. Of note, many voxels in frontal ECN core nodes were ‘low confidence’ voxels. Our conclusions of ECN connectivity are therefore made with lower confidence than our inferences about the DMN and SN connectivity changes. See Figure 3 for a summary of BPI results. Network component maps, group statistic t and *P* value maps, concatenated component beta maps, and LPO maps are available at: https://osf.io/9r3um/.

## Discussion

To the best of our knowledge, this is the first study to investigate the triple network within a pharmacological paradigm administering both 10 mg of oral yohimbine and/or 10 mg of hydrocortisone in a group of healthy male volunteers. Participants were randomly assigned to one of four treatment groups (placebo + placebo; yohimbine + placebo; placebo + hydrocortisone; yohimbine + hydrocortisone) and underwent resting state scanning at a timeframe when slow genomic actions of cortisol begin to unfold (two hours after drug intake). In line with previous predictions of this timeframe, we expected pharmacological elevations of noradrenaline and cortisol (especially in their combination) to lead to delayed increases in resting state connectivity within the ECN and decreases of such connectivity within the SN (when compared to placebo). Because prior findings from the DMN were inconclusive, and its connectivity could have plausibly been up- or downregulated following exposure to stress neuromodulators, we explored DMN connectivity in both directions. We found no connectivity differences between treatment groups within any of the three networks of interest (both using classic non-parametric hypothesis testing and BPI). This is despite salivary markers of noradrenaline and cortisol both still being significantly elevated at the time of resting state scanning, in those groups which received the corresponding medications (compared to baseline and compared to groups which did not receive the medication).

Our results contrast with predictions made by Hermans *et al.,* (2014) who, on the basis of a large number of behavioral and activation based paradigms, predicted the stress recovery phase to be characterized by SN down- and ECN upregulation (Hermans *et al.*, 2014). These predictions were corroborated by findings of reduced SN connectivity and increased ECN-cerebellum connectivity 90 minutes following psychosocial stressor onset (van Leeuwen *et al.*, 2021). Our findings are also somewhat incongruent with seed-based paradigms reporting increased SN-DMN connectivity 60 minutes (Veer *et al.*, 2011) and 120 minutes (Vaisvaser *et al.*, 2013) following psychosocial stress, suggestive of a SN recruitment of the DMN during stress recovery (or the reverse). In the following, we will discuss these discrepancies in light of differences between psychosocial *vs* pharmacological paradigms, task *vs* resting paradigms, seed-based *vs* whole-network connectivity, as well as early *vs* late neuromodulatory effects.

Because we did not find triple network changes in the presence of significantly elevated levels of noradrenaline and cortisol, this could indicate that the two stress neuromodulators alone do not recruit networks in the way previously proposed. A possible line of reasoning for such interpretation could be that a majority of previous evidence came from approaches implementing psychosocial stress induction (rather than pharmacological manipulations). Prior work has demonstrated that these stress induction procedures may have led to paradigm-specific confounds, as studies using the same paradigm yielded more congruent results than studies using different paradigms (van Oort *et al.*, 2017). For example, considering the DMN’s role in internal cognition and rumination (Zhou *et al.*, 2020; Menon, 2023), it is conceivable that social evaluative feedback inherent to many psychosocial stress paradigms may have led to (prolonged) periods of self-referential thought, and thereby to prolonged changes in DMN connectivity found in previous studies. Because such social evaluative elements were absent from our paradigm, this may explain why the DMN was unaffected in our study. Similarly, psychosocial paradigms often involve more complex cognitive operations in stress conditions, as compared to control conditions (e.g. subtracting in steps of 13 *vs* adding in steps of seven). Therefore, previous studies may have possibly conflated ECN effects of stress with those of cognitive load (van Oort *et al.*, 2017). Since our design is unlikely to have induced systematic group differences in cognitive load, this could explain why we found no changes in ECN connectivity following (combined) administration of stress neuromodulators. One possible explanation for our null results therefore is that prolonged network changes found in previous studies may not have been the result of stress per se, but a by-product of psychosocial stress induction.

However, the flip side of interpretations offered in the previous paragraph is that pharmacological manipulations cannot really be considered stress. Although increasing stress neuromodulators may arguably provide a cleaner estimate of related network changes without confounds of psychosocial stress induction, and investigating both noradrenaline and cortisol may be more ecologically valid than only investigating cortisol, such manipulation brings with it its own shortcomings. The stress response is associated with cascades of hormones and neuropeptides, whose actions are interwoven in complex preparatory and suppressive ways. This includes monoamines, such as serotonin and dopamine, neuropeptides such as vasopressin, orexin and corticotropin-releasing hormone, as well as downstream adrenocorticotropic hormone (Joëls and Baram, 2009). Without the context of these other neuromodulators, rises in noradrenaline and cortisol may be ‘interpreted’ differently by the brain, as both have many physiological roles outside of their involvement in the stress response (O’Donnell *et al.*, 2012; McEwen, 2019). For example, cortisol levels akin to stress are present in the morning in order to introduce the active phase, in appetitive and rewarding situations, as well as during sex and vigorous physical activity (Koolhaas *et al.*, 2011; Joëls, 2018). What makes an experience stressful in a psychological sense has been related to the degree to which it is interpreted as uncontrollable and unpredictable (Koolhaas *et al.*, 2011; Sandi, 2013). While for some people the scanning environment may have been stressful, it is likely that many of our participants did not experience the setup as unpredictable or uncontrollable (they were briefed about the procedure, received verbal check-ins after every scan, and were told they could terminate the experiment at any time). This could then disguise differences in brain activity, as is suggested by findings from yoked (conditioning) paradigms showing that brain activity in threat-related areas is robustly reduced when stressors are predictable and/or controllable (Wood *et al.*, 2015; Limbachia *et al.*, 2021). Together, this offers another reason why networks might not have been differentially affected across treatment groups. Participants receiving yohimbine and/or hydrocortisone might not have been sufficiently stressed and therefore recruitment of stress-related networks may have been dampened or absent.

Though these offer plausible explanations for differences between our results and those of other groups, it is important to note a few other systematic differences, which complicate drawing definite conclusions. Next to psychosocial stress paradigms discussed in the previous paragraphs, there is a body of pharmacological studies which have investigated the stress recovery phase by examining the activity of specific brain regions during task performance (e.g. Henckens *et al.*, 2010, 2011, 2012). Our paradigm also differs from these approaches with its focus on resting connectivity rather than activity. Resting state approaches have considerable advantages in that they are better equipped than other (blocked) designs to characterize diffuse and temporally unfolding brain states, like those associated with stress, and circumvent conflation of task-related and stress-related activity (van Oort *et al.*, 2017). However, these advantages coincide with the disadvantage of limiting our ability to make inferences about the brain’s response to the environment. Although there should be correspondence between task-related and resting state connectivity within networks (Smith *et al.*, 2009; Mennes *et al.*, 2010), it is conceivable that compared to task-related designs, networks at rest were insufficiently activated to reveal differences between groups. This may not be so problematic for the DMN, since it is known to be active at rest (Menon, 2023), but it may have been relevant for more task-related networks like the ECN and SN. For example, the dorsolateral prefrontal cortex (a core ECN node) showed heightened activity during working memory performance 240 minutes following hydrocortisone administration (Henckens *et al.*, 2011), while the amygdala (SN node) showed dampened reactivity to positive emotional faces 285 minutes following hydrocortisone administration (Henckens *et al.*, 2010). The absence of network engagement in our study may therefore also be explained by the absence of a task to engage network nodes. This could be because at a cellular level, glucocorticoids are known to act in a conditional manner, that is, they often exert their influence only after cells are shifted from their resting potential (which would be expected to be more often the case when regions are active rather than at rest) (de Kloet *et al.*, 2018). Nevertheless, there has been at least one previous investigation which found SN and ECN-related changes during rest, however they also differed in timepoint (90 minutes following stressor onset) and paradigm (psychosocial stress rather than pharmacological manipulation) (van Leeuwen *et al.*, 2021).

Another key differentiating feature of our study compared to other paradigms concerns the analysis methods employed. We only know of one study which adopted a whole network approach at a comparable timepoint (van Leeuwen *et al.*, 2021). All other approaches investigated either activity of single regions, as described in the previous paragraph, or connectivity of individual seed regions to the rest of the brain. For example, by selecting seeds in the amygdala (SN) or hippocampus and posterior cingulate cortex (DMN), two previous approaches found increased SN-DMN connectivity 60 and 120 minutes after stress induction (Veer *et al.*, 2011; Vaisvaser *et al.*, 2013). Seed-based approaches are more sensitive to specific changes but potentially omit other relevant changes, which can hamper inter-study comparison (van Oort *et al.*, 2017). For example, selecting an amygdala seed, Veer *et al.,* (2011) found increased connectivity to three regions within the DMN (posterior cingulate cortex, precuneus, and medial prefrontal cortex) 60 minutes following psychosocial stress induction (Veer *et al.*, 2011). By inference, this could also hint at greater within-DMN connectivity, however, this was not tested directly and we therefore cannot compare it to our results.

Finally, because levels of both salivary alpha amylase and cortisol were still significantly elevated at the time of resting state scanning, it could be argued that our timepoint may have conflated early and late effects. With our timing mimicking the late stress phase and our saliva results indicating neuromodulatory levels akin to the early phase, it could be argued that participants may have exhibited opposite network patterns associated with the two phases (Hermans *et al.*, 2014), leading to network effects cancelling each other out. For the SN and DMN, BPI results render this interpretation unlikely because the majority of (core) voxels were ‘no change voxels’ with 95% of ESs within the ROPE (Figure 2). For the ECN however, frontal core nodes included many ‘low confidence’ voxels, meaning that ESs were more widely spread to the negative and positive sides of the ROPE—which could be an indication for opposing network changes. However, as the majority of voxels in the ECN contrast still were classified as ‘no change voxels’, this interpretation is only tentative. Future studies may further increase the interval between medication intake and network analyses in order to exclude the possibility of phase mixing.

In summary, we found no differences in resting SN, ECN, or DMN connectivity two hours following pharmacological elevation of noradrenaline and/or cortisol (as compared to the placebo group). This is incongruent with predictions of an ECN up- and SN downregulation in the aftermath of stress, as well as with findings of increased connectivity between nodes of the SN and DMN following psychosocial stress. These discrepancies may have arisen because previous studies have often implemented psychosocial stress induction, which differs from pharmacological approaches in two important ways. While on one hand, pharmacologically elevating stress neuromodulators may have excluded confounds related to psychosocial stress induction, on the other, it cannot really be considered stress in both a biological (missing other neuromodulators) and psychological sense (missing the subjective experience of unpredictability and uncontrollability). Other reasons include networks being insufficiently activated to reveal stress-related effects in the absence of a task and previous studies adopting seed-based rather than whole-network analysis methods.

### Strengths and limitations

To the best of our knowledge, this is the first study to investigate triple network changes within a pharmacological paradigm administering both yohimbine and hydrocortisone in a relatively large sample of healthy males. Investigations of the recovery phase overall are sparse and so far have rarely implemented a whole network perspective (possibly the result of publication bias). A limitation of our experimental setup was the seamless transition from the dotprobe task to the resting state scan. Task-related brain states have been shown influence on subsequent resting-state dynamics (Tailby *et al.*, 2015). Therefore, this setup may have led to more homogeneous resting-state dynamics across groups, hindering the detection of potentially subtle differences between them. Another limitation concerns the fact that salivary levels of alpha amylase and cortisol were still significantly elevated around the time of resting state scanning. Our results therefore cannot fully distinguish between early and late neuromodulatory phases, as well as between genomic and non-genomic cortisol effects. A final limitation is the investigation of only male participants, which limits extension of findings to females. Considering that there are robust sex differences in stress-related neural and behavioral changes (Bangasser *et al.*, 2019; Goldfarb *et al.*, 2019), directly comparing network responses of males and females would be an interesting endeavor for future research.

### Future direction

Although triple network theories of stress have gained a lot of attention in the last decade (Hermans *et al.*, 2014; van Oort *et al.*, 2017), only a few studies investigated networks across time (e.g. Vaisvaser *et al.*, 2013; van Leeuwen *et al.*, 2021). To broaden our understanding of within- and between-network dynamics related to stress, future research should aim to implement repeated measures designs for at least 2 hours following stressor onset, in order to capture recovery processes. Although we believe that resting-state data have special utility in repeated measures designs, excluding task-related activity and learning effects, implementing tasks at key timepoints could broaden our understanding of stress-related network functioning. For example, the stress recovery phase is associated with adaptive increases in emotion regulation, contextualization, and consolidation of memories (Langer *et al.*, 2021; Schwabe *et al.*, 2022). All these functions are associated with the DMN (Menon, 2023), making task-related DMN activation during stress recovery a highly interesting endeavor for future research. Finally, to parse apart effects related to different stress induction procedures from those related to stress, it would be interesting to meta-analytically investigate different kinds of experimental paradigms, in order to identify convergent activity and connectivity.

### Conclusions

We found no differences in within network connectivity of the SN, DMN and ECN two hours following pharmacological elevation of stress neuromodulators noradrenaline and/or cortisol (as compared to placebo). While this could hint at previous studies of this timepoint being confounded by elements of psychosocial stress induction, substantial heterogeneity in study designs makes it difficult to draw definite conclusions. We suggest future research to systematically investigate differences between paradigms, in order to better distinguish between stress-related and paradigm-related effects.

## Supplementary Material

nsad073_Supp

## Data Availability

Data cannot be made publicly available as participants were not explicitly asked and therefore did not consent to making their data public. All reported statistical maps are available at https://osf.io/9r3um/.
