# Supplementary material for: No changes in triple network engagement following (combined) noradrenergic and glucocorticoid stimulation in healthy men
Source: Soc Cogn Affect Neurosci. 2023 Dec 20;19(1):nsad073. doi: 10.1093/scan/nsad073 (PMC10868128; doi:10.1093/scan/nsad073)
Supplement: nsad073_Supp [file nsad073_supp.zip › scan-23-073-File002.docx]

**Supplementary Material**

[Network components 1](#_Toc142463203)

[Bayesian parameter inference 1](#_Toc142463204)

[Heart rate and blood pressure 2](#_Toc142463205)

[Group comparison results using non-parametric testing 4](#_Toc142463206)

[References 5](#_Toc142463207)

# Network components

Supplementary Figure 1.

# Bayesian parameter inference

Bayesian parameter inference (BPI) comprises of two steps: parameter estimation and inference. Each will be elaborated on in the following sections.

***Parameter estimation***

Bayesian parameter estimation comprises of finding the posterior probability distribution of the experimental effect at each voxel, based on prior beliefs about the effect and the obtained data (Masharipov *et al.*, 2021). One of the main challenges of Bayesian statistics is choosing appropriate prior distributions. However, as neuroimaging data are organized hierarchically, the individual-level beta estimates obtained for each voxel can be utilized to estimate the prior variance in a parametric empirical Bayes (PEB) approach (Friston and Penny, 2003; Masharipov *et al.*, 2021). In our design, individual-level beta estimates were outputs from the second stage of the dual regression, which represent how strongly each voxel contributed to the group-level network of interest (Nickerson *et al.*, 2017). The PEB then specified the same Gaussian ‘global shrinkage prior’ for each voxel. This prior has a zero mean (assuming that a global experimental effect is absent – with some voxels having high and others having low beta estimates), and a variance equal to one standard deviation of beta estimates across all voxels (Masharipov *et al.*, 2021). This prior was then updated by the data obtained at each voxel individually (the actual distribution of beta estimates at a given voxel across volumes), to obtain the posterior probability distribution of the beta estimate at that voxel. If the variance around obtained beta estimates in a given voxel was large, then the resulting posterior would ‘shrink’ more towards the prior with a zero mean. If the beta variance of the voxel was small, the posterior would ‘shrink’ less and its mean would remain farther away from zero (Masharipov *et al.*, 2021). The result is a posterior probability map (PPM), indicating the posterior probability distribution of each voxels beta estimate.

***Parameter inference***

Next, the group-level posterior probability distributions of the experimental effect were determined at each voxel using linear contrast weight vectors (comparing the group receiving both yohimbine and hydrocortisone with the placebo group in all three networks). BPI is based on the posterior probability of finding the experimental effect within or outside the region of practical equivalence (ROPE) to the null value. The ROPE is defined by the effect size (ES) threshold [-γ; γ]. For group level BPI, the default ES threshold is one prior SD of the group experimental effect, which we also used; Masharipov et al., 2021). We used the ROPE-only decision rule with posterior probability threshold *P_thr_* = 95%, which is equal to the Log Posterior Odds (LPO) > 3 (Masharipov *et al.*, 2021). This way, voxels with 95% of their posterior probability distributions beyond γ were classified as showing ‘increased network strength’, those with 95% of their posterior probability distribution below -γ were classified as showing ‘decreased network strength’, and those 95% of their posterior probability distribution within the ROPE were considered to show ‘no network changes’. If none of these criteria were met, voxels were considered ‘low confidence voxels’ for which our data are insufficient to make inferences.

# Heart rate and blood pressure

To determine the effects of group assignment on heart rate, systolic- and diastolic blood pressure, three mixed repeated measures ANOVAs were performed with time as the within-subjects factor and yohimbine (yes/no) and hydrocortisone (yes/no) as between-subjects factors. There was a significant effect of yohimbine on systolic blood pressure. Main effect yohimbine: F(1, 158) = 12.23, *p* = .001, *η^2^* = .07; and time x yohimbine interaction: F(3.46, 547.73) = 4.19, *p* = .004, *η^2^* = .02. Bonferroni corrected post hoc t-tests (one tailed) revealed that groups which received yohimbine, as compared to those who did not, showed higher mean systolic blood pressure at time +75 (t(163) = 3.30, *p* = .005, *d* = .51), time +155 (t(149.82) = 2.95, *p* = .017, *d* = .46), and time +170 (t(160) = 3.49, *p* = .003, *d* = .55). There was no significant effect of hydrocortisone on systolic blood pressure: F(1, 158) = .09, *p* = .75, *η^2^* = .00.

Similarly, when examining diastolic blood pressure, there was a significant effect of yohimbine (but not hydrocortisone). Main effect yohimbine: F(1, 158) = 6.28, *p* = .01, *η^2^* = .03 and time x yohimbine interaction: F(3.47, 548.41) = 3.00, *p* = .024, *η^2^* = .01. Bonferroni corrected post hoc t-tests (one tailed) revealed that those groups who had received yohimbine, compared to those who did not, had higher mean diastolic blood pressure at time +75 (t(163) = 3.37, *p* = .004, *d* = .52). There was no significant effect of hydrocortisone on diastolic blood pressure: F(1, 158) = .42, *p* = .51, *η^2^* = .00.

Finally, there was no effect of either yohimbine or hydrocortisone on heart rate. Main effect yohimbine: F(1, 158) = .23, *p* = .62, *η^2^* = .00 and main effect hydrocortisone: F(1, 158) = .09, *p* = .76, *η^2^* = .00.

Overall, these results indicate that groups who have received yohimbine (as compared to those who did not) showed significant increases in systolic blood pressure at the timepoints following yohimbine administration (+75, +155, +170). Diastolic blood pressure of yohimbine-receiving groups was elevated just before entering the scanner (at time +75). Hydrocortisone had no significant effects on neither systolic nor diastolic blood pressure over time. Finally, heart rate was unaffected by drug administration overall. See Supplementary Figure 2 for an overview of treatment effects on blood pressure and heart rate.

Supplementary Figure 2.

# Group comparison results using non-parametric testing

Supplementary Figure 3.

Supplementary Table 1.

# References

Friston, K.J., Penny, W. (2003). Posterior probability maps and SPMs. *NeuroImage*, **19**, 1240–49

Masharipov, R., Knyazeva, I., Nikolaev, Y., et al. (2021). Providing evidence for the null hypothesis in functional magnetic resonance imaging using group-level bayesian inference. *Frontiers in Neuroinformatics*, **15**, 738342

Nickerson, L.D., Smith, S.M., Öngür, D., et al. (2017). Using dual regression to investigate network shape and amplitude in functional connectivity analyses. *Frontiers in Neuroscience*, **11**
