# Supplementary material for: No changes in triple network engagement following (combined) noradrenergic and glucocorticoid stimulation in healthy men
Source: Soc Cogn Affect Neurosci. 2023 Dec 20;19(1):nsad073. doi: 10.1093/scan/nsad073 (PMC10868128; doi:10.1093/scan/nsad073)
Supplement: nsad073_Supp [file nsad073_supp.zip › scan-23-073-File010.docx]

| Supplementary Table 1. Group comparison results for each network | | | | | | |
| --- | --- | --- | --- | --- | --- | --- |
| Networks, contrasts, and cluster locations | Cluster size (voxels) | Peak  t values | Peak  1-p values | MNI coordinates | | |
|  |  |  |  | x | y | z |
| SN |  |  |  |  |  |  |
| PLA > YOH | - | - | - | - | - | - |
| PLA > HYD | - | - | - | - | - | - |
| PLA > YOH & HYD | - | - | - | - | - | - |
| DMN |  |  |  |  |  |  |
| YOH > PLA | - | - | - | - | - | - |
| HYD > PLA | - | - | - | - | - | - |
| YOH & HYD > PLA | - | - | - | - | - | - |
| PLA > YOH | - | - | - | - | - | - |
| PLA > HYD | - | - | - | - | - | - |
| PLA > YOH & HYD | - | - | - | - | - | - |
| ECN |  |  |  |  |  |  |
| YOH > PLA | - | - | - | - | - | - |
| HYD > PLA | - | - | - | - | - | - |
| YOH & HYD > PLA | - | - | - | - | - | - |
| L Cerebral White Matter (91.1%) | 689 | 4.80 | 0.995 | 66 | 41 | 50 |
| L Cerebral Cortex (82.6%) | 455 | 4.61 | 0.996 | 50 | 52 | 57 |
| R Cerebral White Matter (97.3%) | 453 | 4.23 | 0.995 | 29 | 33 | 44 |
| R Cerebral Cortex (99.3%) | 409 | 4.35 | 0.992 | 43 | 54 | 59 |
| R Cerebral White Matter (83.1%) | 299 | 4.20 | 0.993 | 36 | 47 | 55 |
| R Cerebral Cortex (74.8%) | 122 | 4.88 | 0.992 | 41 | 82 | 50 |
| L Cerebral White Matter (100.0%) | 97 | 4.52 | 0.992 | 54 | 63 | 53 |
| R Cerebral White Matter (45.9%) | 95 | 4.44 | 0.993 | 31 | 46 | 30 |
| R Thalamus (68.3%) | 94 | 3.60 | 0.990 | 40 | 48 | 36 |
| R Cerebral White Matter (100.0%) | 56 | 3.81 | 0.988 | 27 | 47 | 36 |
| R Cerebral Cortex (11.0%) | 43 | 4.36 | 0.990 | 39 | 36 | 31 |
| L Cerebral White Matter (60.0%) | 26 | 3.97 | 0.989 | 53 | 43 | 62 |
| L Cerebral White Matter (100.0%) | 20 | 3.72 | 0.988 | 58 | 71 | 47 |
| R Cerebral Cortex (60.0%) | 15 | 3.97 | 0.988 | 44 | 22 | 39 |
| L Cerebral Cortex (78.8%) | 14 | 3.28 | 0.987 | 66 | 56 | 62 |
| L Cerebral Cortex (82.1%) | 13 | 4.02 | 0.989 | 72 | 57 | 61 |
| Abbreviations: DMN = Default Mode Network, ECN = Executive Control Network, SN = Salience Network, PLA = Placebo, YOH = Yohimbine, HYD = Hydrocortisone, MNI = Montreal Neurological Institute, L = Left, R = Right. All results are TFCE corrected for multiple comparisons across voxels within networks and Bonferroni corrected (p = 0.05/3 = 0.016) for multiple comparisons across networks (as prescribed in <https://fsl.fmrib.ox.ac.uk/fsl/fslwiki/DualRegression/UserGuide#Multiple-comparison_correction_across_all_RSNs>). Only clusters > 10 voxels are displayed. | | | | | | |
