# Supplementary figures and images for: No changes in triple network engagement following (combined) noradrenergic and glucocorticoid stimulation in healthy men
Source: Soc Cogn Affect Neurosci. 2023 Dec 20;19(1):nsad073. doi: 10.1093/scan/nsad073 (PMC10868128; doi:10.1093/scan/nsad073)

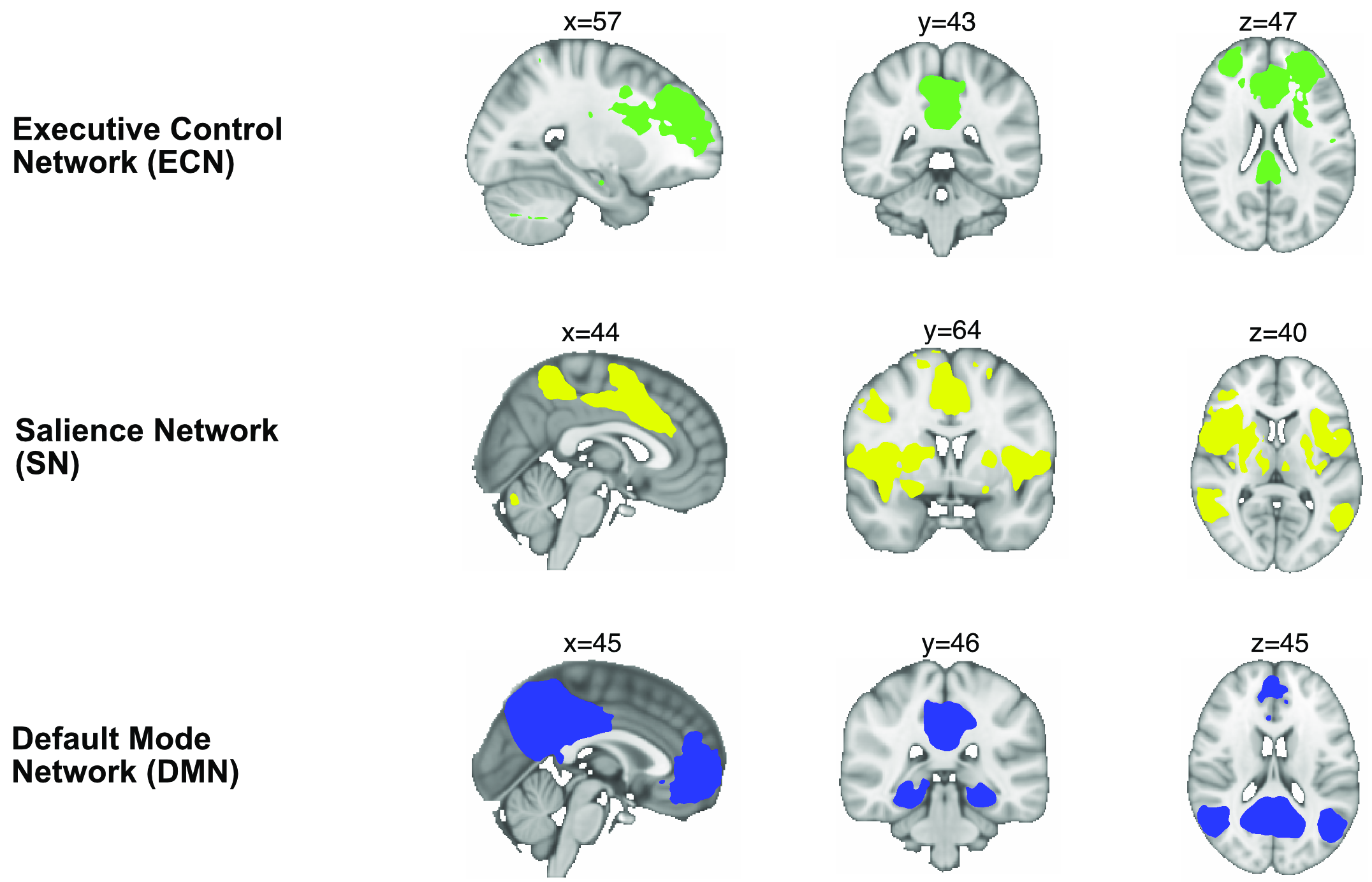

Supplement: nsad073_Supp [file nsad073_supp.zip › scan-23-073-File006.tiff]

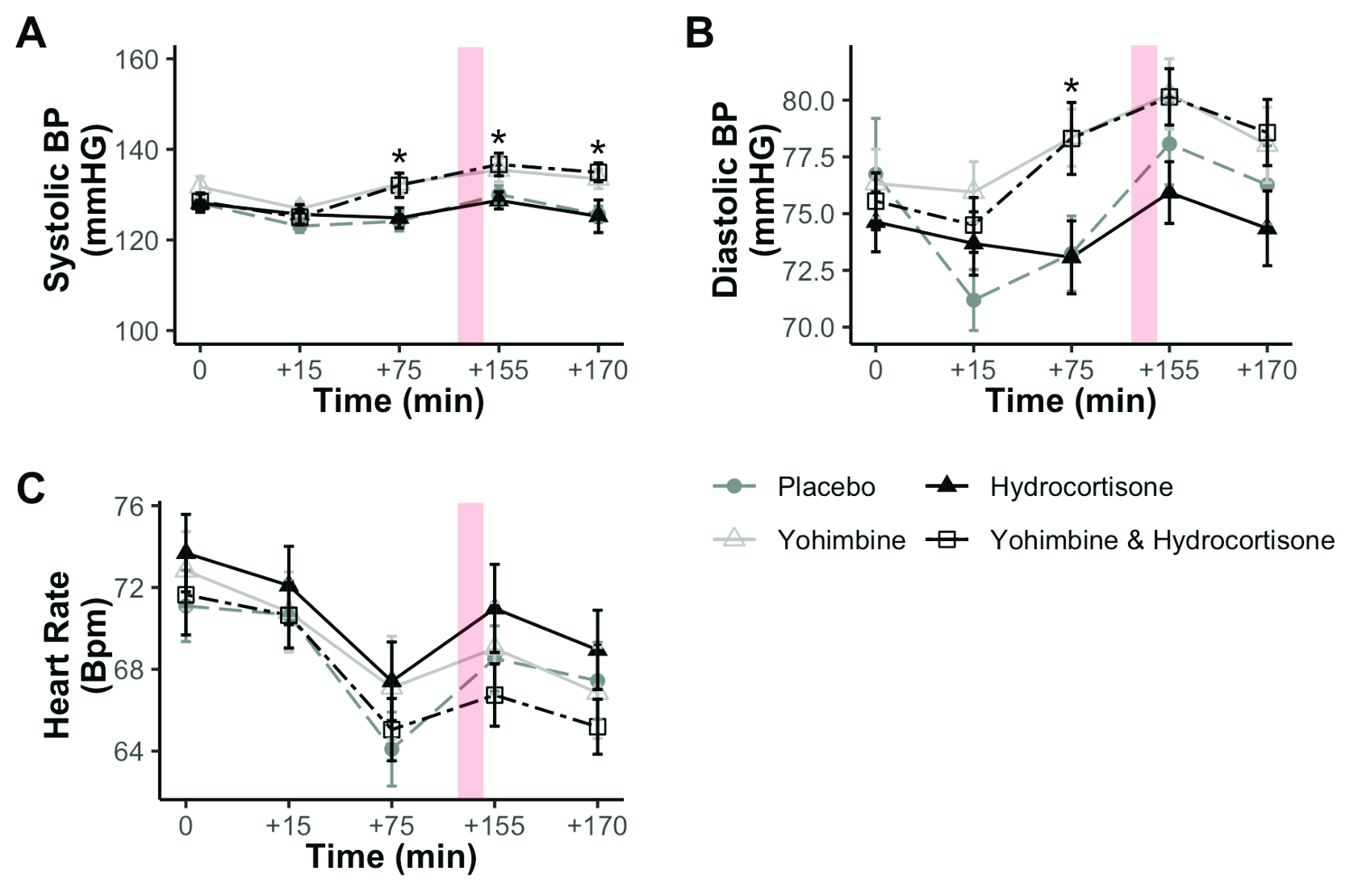

Supplement: nsad073_Supp [file nsad073_supp.zip › scan-23-073-File007.tiff]

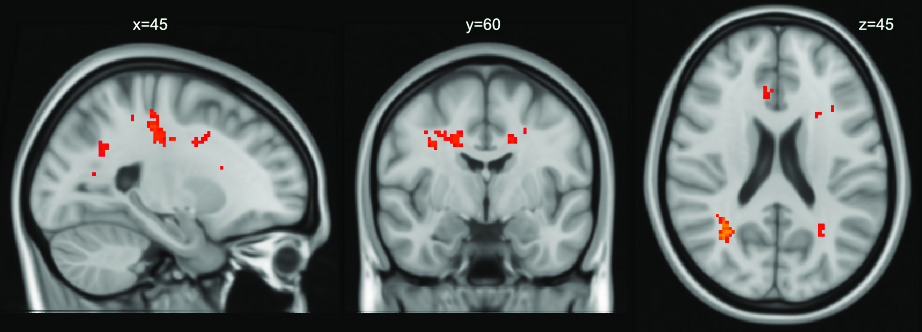

Supplement: nsad073_Supp [file nsad073_supp.zip › scan-23-073-File008.tiff]
